# Prevalence of Undiagnosed Monkeypox Virus Infections during Global Mpox Outbreak, United States, June–September 2022

**DOI:** 10.3201/eid2911.230940

**Published:** 2023-11

**Authors:** Faisal S. Minhaj, Vijay Singh, Stephanie E. Cohen, Michael B. Townsend, Hyman Scott, John Szumowski, C. Bradley Hare, Pallavi Upadhyay, Jairus Reddy, Barbara Alexander, Nicolle Baird, Terese Navarra, Lalita Priyamvada, Nhien Wynn, William C. Carson, Solomon Odafe, Sarah Anne J. Guagliardo, Emily Sims, Agam K. Rao, Panayampalli S. Satheshkumar, Paul J. Weidle, Christina L. Hutson

**Affiliations:** Centers for Disease Control and Prevention, Atlanta, Georgia, USA (F.S. Minhaj, M. Townsend, N. Baird, T. Navarra, L. Priyamvada, N. Wynn, W.C. Carson; S. Odafe, S.A.J. Guagliardo, E. Sims; A.K. Rao, P.S. Satheshkumar, P.J. Weidle, C.L. Hutson);; HealthTrackRx, Denton, Texas, USA (V. Singh, P. Upadhyay, J. Reddy, B. Alexander);; San Francisco Department of Public Health, San Francisco, California, USA (S.E. Cohen, H. Scott);; University of California San Francisco and Zuckerberg San Francisco General Hospital, San Francisco (J. Szumowski);; Kaiser Permanente Northern California, San Francisco (C.B. Hare)

**Keywords:** Monkeypox virus, Mpox, orthopoxvirus, IgM, PCR, undiagnosed, viruses, United States

## Abstract

Since May 2022, mpox has been identified in 108 countries without endemic disease; most cases have been in gay, bisexual, or other men who have sex with men. To determine number of missed cases, we conducted 2 studies during June–September 2022: a prospective serologic survey detecting orthopoxvirus antibodies among men who have sex with men in San Francisco, California, and a retrospective monkeypox virus PCR testing of swab specimens submitted for other infectious disease testing among all patients across the United States. The serosurvey of 225 participants (median age 34 years) detected 18 (8.0%) who were orthopoxvirus IgG positive and 3 (1.3%) who were also orthopoxvirus IgM positive. The retrospective PCR study of 1,196 patients (median age 30 years; 54.8% male) detected 67 (5.6%) specimens positive for monkeypox virus. There are likely few undiagnosed cases of mpox in regions where sexual healthcare is accessible and patient and clinician awareness about mpox is increased.

Since May 2022, monkeypox virus (MPXV) infections have been detected in 104 countries without endemic disease. Most cases have been among gay, bisexual, or other men who have sex with men (MSM). Because lesions commonly occur on the genitals, mpox was most frequently diagnosed in clinics conducting sexually transmitted infection (STI) screening (*1*). The diagnosis can be challenging because the mpox rash has been confused with STIs (e.g., herpes simplex virus infection and syphilis), hand-foot-and-mouth disease, varicella zoster virus infection, and even arthropod bites (*2*–*4*). In addition to cases being undiagnosed because of diminished clinical suspicion, some cases may have been undiagnosed if patients did not seek care (i.e., because the symptoms were mild and self-limiting or because of poor access to a medical provider). As the global outbreak continued, public health authorities continued to increase awareness of mpox. However, clinicians and public health authorities were concerned that if a high number of cases were missed, the outbreak would be difficult to control. To determine the number of undiagnosed MPXV infections in the United States, we conducted 2 studies during June–September 2022: a prospective serologic surveillance study among MSM who sought sexual health services in San Francisco, California, USA, and a retrospective study of molecular testing of specimens tested for other infectious diseases linked to specific codes from the International Classification of Diseases, 10th Revision, Clinical Modification (ICD-10-CM), among all populations. Each study used specimens collected during the peak of the outbreak. Our studies were reviewed by the Centers for Disease Control and Prevention (CDC) and were conducted consistent with applicable federal law and CDC policy (e.g., 45 C.F.R. part 46.102(l)(2), 21 C.F.R. part 56; 42 U.S.C. §241(d); 5 U.S.C. §552a; 44 U.S.C. §3501 et seq.).

## Methods

### Prospective Serologic Survey

For the primary recruitment sites for this serologic survey, we selected 3 prominent sexual health clinics (clinics A, B, C) in San Francisco that regularly treat MSM and 1 research clinic in San Francisco (clinic D). Those 4 private and publicly funded clinics encompass an estimated 20,000 MSM patients of varying socioeconomic status, insured rates (2%–85% private, 14%–92% public, 0–40% uninsured), and races and ethnicities within the San Francisco Bay area. Patients entering the 3 sexual health clinics during June 28–August 26, 2022, were given an informational flier in English or Spanish containing a QR code that directed interested patients to a survey to self-screen for inclusion. The flier also stated that participation was voluntary and the decision to enroll would not in any way affect their medical care. Study inclusion was limited to patients who self-reported that they did not have symptoms of mpox (e.g., rash, fever, lymphadenopathy), had never received an mpox diagnosis, were 18–50 years of age (the upper age limit was set to exclude childhood smallpox vaccination in the United States), and did not have a history of smallpox or mpox vaccination. Because most cases detected at that point in the outbreak were in MSM, and to ensure sufficient participation among this population at high risk for mpox, we also excluded cisgender women and persons who did not identify as ever having had male-to-male sexual contact. Participants at clinic D were recruited by a query into the electronic medical record system from HIV and HIV pre-exposure prophylaxis registries; a subset of MSM patients 18–50 years of age were sent an invitation to participate. At clinic arrival, those participants were given the same survey to self-screen. All participants who completed the self-screening questionnaire and were eligible for study inclusion, then completed a brief 7-question electronic survey that asked about factors thought to be associated with risk for mpox during the initial stage of the outbreak that could affect public health action (e.g., travel and exposure history within the past 90 days) (Appendix 1) (*3*). We collected 5 mL of blood from each participant who completed the questionnaire. Peak IgM is detected 2–3 weeks and IgG 3–5 weeks after exposure to an orthopoxvirus (including primary vaccination with ACAM2000 [second-generation live vaccinia virus vaccine] and JYNNEOS [third-generation live, non-replicating, modified vaccinia Ankara Bavarian Nordic vaccine; https://jynneos.com]), and convalescence has been documented at 7–14 weeks after exposure. Orthopoxvirus IgM is reliably detected 4–56 days and IgG >8 days after rash onset (*5*). Because IgG persists for several years after orthopoxvirus exposure (*6*), we chose IgG as the initial screening tool to detect any past orthopoxvirus exposure. To detect recent exposure, we tested positive IgG specimens for IgM. We separated serum by centrifugation, aliquoted the samples, and sent them to CDC for ELISA analysis of orthopoxvirus IgG and, if positive, IgM.

### Retrospective Molecular Testing

During the multinational outbreak that began in 2022, mpox was diagnosed by nonvariola orthopoxvirus- and MPXV-specific real-time PCR tests of lesion swab samples (*7*,*8*). Before an mpox-specific diagnosis code (B.04) was established, clinical diagnoses and testing were documented with ICD-10-CM codes representing broad symptoms of infection, which were used as a surveillance tool for early identification of potentially undiagnosed infections similar to other diseases (*9*,*10*). To evaluate the presence of MPXV within specimens received for other testing, CDC partnered with HealthTrackRx, a private laboratory that receives specimens from a variety of clinics across the United States for infectious disease testing. During June 1–September 2, 2022, CDC deidentified and tested lesion swab specimens associated with ICD-10-CM codes for genital lesions, herpes simplex virus infection, inflammation of the genital region, skin rash, and others that may overlap with symptoms of mpox (Appendix 2) for presence of MPXV DNA by using a clade II–specific PCR (*8*). After June 27, 2022, HealthTrackRx validated its own mpox clade II–specific assay (*8*) and continued to test specimens for MPXV that fit the ICD-10-CM codes (Appendix 2). No specimens were excluded; only basic demographic and geographic data and pertinent ICD-10-CM codes that may be associated with mpox were available from the initial test request from the submitting clinician. No information about sexual history was included.

## Results

### Prospective Serologic Survey

During the study period, ≈8,670 patients were seen at clinics A, B, and C, of which 3,832 (44.2%) were MSM, 18–50 years of age, and may have been eligible for participation. An estimated 6,000 persons from clinic D were eligible for study participation, and 2,400 (40%) were sent an invitation to participate. A total of 398 patients started the survey. Of 358 (87.4%) participants who completed the survey, 133 were excluded for not self-identifying as having male-to-male sexual contact (n = 67), reporting previous receipt of smallpox or mpox vaccination (n = 41), being >50 years of age (n = 18), or reporting a past diagnosis of mpox (n = 7). We collected serum samples from the final sample size of 225 participants. Participant median age was 34 (interquartile range [IQR] 29–42) years. Most (52.9%) eligible participants were non-Hispanic White, and most (87.1%) reported sexual orientation as gay (Tables 1, 2). Twenty-six (11.6%) participants reported known contact with someone with mpox. Recent travel (previous 3 months) was reported by 77 (34.2%); among the 67 who reported a location, 38 (56.7%) had traveled in the United States, 17 (25.4%) to Europe, and 13 (19.4%) to other countries within the Americas. A total of 130 (57.8%) participants had attended a large private or public event (e.g., festivals, parades, weddings, clubs, sex parties). Most (203, 91.2%) participants had >1 sexual contact in the previous month, among which 68 (30.2%) had >5 partners. A total of 65 (28.9%) participants had an immunocompromising condition, most commonly HIV (89.2%; n = 58). Of those who reported HIV, 8 (13.8%) reported a CD4 count <200 cells/mm^3^ and 9 (15.5%) reported a viral load >200 copies/mL. Among the 47 (20.9%) who reported being ill in the previous 3 months, the most common signs/symptoms were cough, rhinorrhea, sore throat, fever, and chills (participants could report >1 sign/symptom).

**Table 1:** Demographics and characteristics of 225 participants in study of prevalence of undiagnosed monkeypox virus infections during the global mpox outbreak, United States, June–September 2022*

Characteristic or demographic	No. (%)
Demographic	
Age, y	34 (29–42)
Race/ethnicity	
Hispanic	42 (18.7)
Non-Hispanic	
Asian	35 (15.6)
Black	20 (8.9)
Native American or Pacific Islander	4 (1.8)
White	119 (52.9)
Prefer not to answer	5 (2.2)
Sexual orientation	
Bisexual	22 (9.8)
Gay	196 (87.1)
Straight	0
A different term	6 (2.7)
Prefer not to answer	1 (0.4)
Clinic	
A	23 (10.2)
B	131 (58.2)
C	52 (23.1)
D	19 (8.4)
Known contact with someone with mpox	26 (11.6)
Travel in the past 3 mo†	77 (34.2)
International	29 (43.3)
Europe	17 (25.4)
Americas	13 (19.4)
Domestic	38 (56.7)
Sex partners in the past 1 mo	
0	22 (9.8)
1–4	135 (60)
5–9	37 (16.4)
10–19	20 (8.9)
>20	11 (4.9)
Attended a large event (e.g., festivals, parades, weddings, clubs, sex parties)	130 (57.8)
Immunocompromising conditions	
Yes	65 (28.9)
HIV	58 (89.2)
CD4 count, cells/mm^3^	
≥200	27 (46.6)
<200	8 (13.8)
Unknown	23 (39.7)
HIV viral load, copies/mL	
≥200	9 (15.5)
<200	34 (58.6)
Unknown	15 (25.9)

*Results of serologic survey (Appendix 1, https://wwwnc.cdc.gov/EID/article/29/11/23-0940-App1.pdf). †Location unknown for 10 persons who had traveled.

**Table 2:** Clinical signs/symptoms reported by participants in study of prevalence of undiagnosed monkeypox virus infections during the global mpox outbreak, United States, June–September 2022*

Acute illness sign/symptoms in past 3 months	No. (%)
Cough	28 (12)
Rhinorrhea	27(11)
Sore throat	23 (10)
Fever	22 (9)
Chills	21(9)
Headaches	20 (8)
Sweats	15 (6)
Malaise	15 (6)
Shortness of breath	12 (5)
Lymphadenopathy	8 (3)
Diarrhea	7 (3)
Rash	6 (3)
Wheezing	5 (2)
Itchiness	4 (2)
Nausea/vomiting	4 (2)
Abdominal pain	4 (2)
Back pain	4 (2)
Rectal bleeding	3 (1)
Stridor	2 (1)
Rectal pain	2 (1)
Eye lesions	1
Pus in stool	1
Penile discharge	1

*Results of serologic survey (Appendix 1).

Of 225 serum samples tested for orthopoxvirus IgG, 18 (8.0%) were also positive and 3 (1.3%) were positive for orthopoxvirus IgM. Those 3 participants were 20–49 years of age. Two patients denied prior smallpox or mpox vaccination; vaccination status for the third patient was unknown. All 3 participants had traveled in the previous 3 months (2 internationally and 1 domestically), 1 reported attending a large event, and 1 reported having had contact with someone with mpox. All 3 participants reported having had 3–20 sex partners within the previous month. Two participants reported signs/symptoms consistent with mpox in the previous 3 months, including rash, diaphoresis, and lymphadenopathy. One participant had well-controlled HIV (CD4 count >200 cells/μL).

### Retrospective Molecular Testing

**Figure 1:**
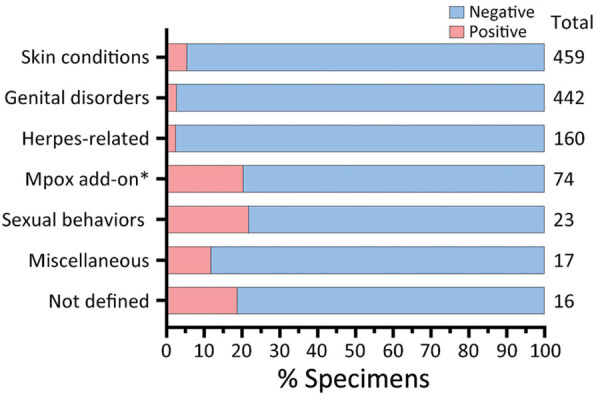
Total numbers and percentages of positive results for specimens tested by monkeypox virus–specific PCR under different code categories from the International Classification of Diseases, 10th Revision, Clinical Modification, United States, June–September 2022. *Mpox add-on is defined as having an initial negative result from other testing, after which a provider requested testing for mpox.

**Figure 2:**
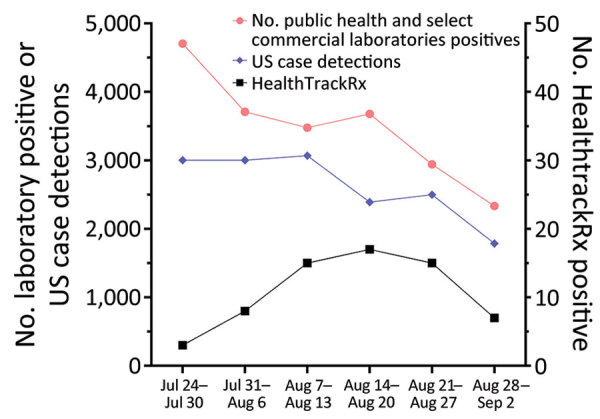
Weekly positive detection of monkeypox virus by PCR testing and US Centers for Disease Control and Prevention case detection (https://www.cdc.gov/ecr/index.html), July 24–September 2, 2022. Results are from public health and select commercial laboratories and HealthTrackRx testing and US case detections. The greater number of laboratory positives than US case detections most likely results from testing of multiple samples from a single patient.

During the study period, MPXV testing was performed for 1,196 patients (median age 30 [IQR 19–46] years); 656 (54.8%) were men. The most common specimen collection sites were arm (24.8%; n = 297), anogenital (18.6%; n = 222), leg (10.1%; n = 121), and unspecified (14.2%; n = 170). The ICD-10-CM codes accompanying specimens were broadly categorized as disorder of the genitals, herpes-related lesions, pruritus, cellulitis, skin conditions, vaginitis, high-risk sexual behavior, mpox, miscellaneous, and not defined. A total of 67 (5.6%) specimens tested positive for MPXV DNA (Figure 1). The dates that the positive specimens had been obtained corresponded to the increase in mpox epidemic curve in the United States (Figure 2). Most MPXV-positive specimens were associated with skin conditions, including ICD-10-CM codes R21 (rash and other nonspecific skin eruption), L98.9 (disorder of skin and subcutaneous tissue, unspecified), L08.89 (other specified local infections of the skin and subcutaneous tissue), and disorders of the genital regions including N48.5 (ulcer of the penis) (Table 3). Among those categories, all specimens with ICD-10-CM codes corresponding to signs/symptoms of pruritis, cellulitis, and vaginitis tested negative for MPXV; no positive specimens were from women. Among the 67 MPXV-positive specimens, 5 (7.3%) ICD-10-CM codes were classified under sexual behavior that places someone at increased STI/HIV risk and 4 (5.8%) under herpes-related lesions. Of the 67 positive specimens, 15 (20.3%) were among 74 specimens that were originally submitted for testing of other infectious organisms but after negative results had been submitted for MPXV testing at provider request.

**Table 3:** International Classification of Diseases, 10th Revision, Clinical Modification codes associated with PCR-positive specimens in study of monkeypox virus infections, United States, June–September 2022

Code	Description
K13.70	Disease of oral mucosa, unspecified
R36.9	Urethral discharge, unspecified
R19.8	Other specified symptoms and signs involving the digestive system and abdomen
R21, R30	Rash and other nonspecific skin eruption, pain associated with micturition
M79.10	Myalgia unspecified site
B08.8, J02.9, R21	Other specified viral infections characterized by skin and mucous membrane lesions
L98.9, N48.9	Disorder of the skin and subcutaneous tissue, unspecified, disorder of penis, unspecified
Z72.52	High-risk homosexual behavior
A63.8, A64	Other specified predominantly sexually transmitted diseases, unspecified sexually transmitted disease
R21, Z11.3	Rash and other nonspecific skin eruption, encounter for screening for infections with a predominantly sexual mode of transmission
L98.9	Disorder of the skin and subcutaneous tissue, unspecified
N48.5	Ulcer of penis
Z20.828	Contact with and (suspected) exposure to other viral communicable diseases
N50.89, R21	Other specified disorders of the male genital organs, rash and other nonspecific skin eruption
A63.0	Anogenital (venereal) warts
A60.1, A63.0	Herpes viral infection of perianal skin and rectum, anogenital (venereal) warts
L08.9	Local infection of skin and subcutaneous tissue, unspecified
N48.5, N50.89, R22.0	Ulcer of penis, other specified disorders of the male genital organs, localized swelling, mass and lump of skin and subcutaneous tissue
B34.9, R50.9, R53.83, Z20.822	Viral infection, unspecified
N48.5, R36.9, Z72.51	Ulcer of penis, urethral discharge, unspecified, high-risk heterosexual behavior
L30.8, R21	Other specified dermatitis, rash and other nonspecific skin eruption
B89, R21	Unspecified parasitic disease, rash and other nonspecific skin eruption
I88.9, N48.89, R36.9	Nonspecific lymphadenitis, unspecified, other specified disorders of penis, urethral discharge, unspecified
K60.2	Anal fissure, unspecified
N48.9, S30.812A	Disorder of penis, unspecified, abrasion of penis
N50.89	Other specified disorders of the male genital organs
L08.89, L98.8	Local infection of skin and subcutaneous tissue, unspecified, other specified disorders of the skin and subcutaneous tissue
Z11.3, Z20.822	Encounter for screening for infections with a predominantly sexual mode of transmission, contact with and (suspected) exposure to other viral communicable diseases
R07.0, R21	Pain in throat, rash and other nonspecific skin eruption
R50.9, Z20.822	Fever, unspecified, contact with and (suspected) exposure to other viral communicable diseases
Z20.2	Contact with and exposure to infections with a predominantly sexual mode of transmission
L30.8	Other specified dermatitis
B04, Z11.3	Monkeypox, encounter for screening for infections with a predominantly sexual mode of transmission
R23.8, Z20.2	Other skin changes, contact with and exposure to infections with a predominantly sexual mode of transmission
A600	Anogenital herpes viral (herpes simplex) infections
B00.2	Herpes viral gingivostomatitis and pharyngotonsillitis
N48.5, Z11.3	Ulcer of penis, encounter for screening for infections with a predominantly sexual mode of transmission
J03.90, K13.79	Acute tonsillitis, unspecified, other lesions of oral mucosa
L03.818	Cellulitis of other sites
L02.818	Cutaneous abscess of other sites

Most specimens received were from Michigan (12.8%), Georgia (12.0%), Colorado (10.4%), and Florida (9.9%); however, the highest proportion of specimens that tested positive for mpox were from Georgia (24.5%, 35 positive), followed by Missouri (25.0%, 5 positive) and Texas (12.9%, 11 positive) (Table 4). Specimens were also tested on the STI and wound infection PCR panels at HealthTrackRx. Among the specimens testing positive for mpox, only 1 tested positive for other etiologies consistent with contamination (*Finegoldia magna*, *Cutibacterium acnes*, and *Peptostreptococcus* spp).

**Table 4:** Positive monkeypox virus PCR detections and case detections by state, United States, June–September 2022*

State	Total tested by HealthTrackRx	MPXV PCR positive, no. (%)	CDC case detections	Mpox cases detected by HealthTrackRx, %
Georgia	143	35 (24.5)	1,602	2.2
Missouri	20	5 (25)	72	6.9
Texas	85	11 (12.9)	2,012	0.5
Nevada	12	1 (8.3)	234	0.4
Illinois	15	1 (6.7)	1,181	0.1
California	31	2 (6.5)	4,389	0
North Carolina	18	1 (5.6)	414	0.2
Michigan	153	7 (4.6)	234	3.0
Florida	119	3 (2.5)	2,269	0.1
Colorado	124	1 (0.8)	268	0.4
Arizona	96	0	386	0
Wisconsin	95	0	74	0
Ohio	90	0	237	0
Arkansas	31	0	40	0
Oklahoma	23	0	29	0
Kentucky	16	0	40	0
Wyoming	16	0	2	0
Alabama	14	0	95	0
Indiana	14	0	195	0
Iowa	10	0	21	0
New Mexico	10	0	35	0
Oregon	10	0	191	0
Idaho	9	0	13	0
Kansas	9	0	7	0
Mississippi	8	0	51	0
Washington	7	0	469	0
Tennessee	6	0	228	0
Maryland	4	0	580	0
New Hampshire	4	0	27	0
New Jersey	2	0	614	0
Rhode Island	2	0	60	0

*CDC, Centers for Disease Control and Prevention; MPXV, monkeypox virus.

## Discussion

A total of 21,798 mpox cases were reported in the United States during the peak of the outbreak, June–September 2022, accounting for 72.0% of the total US cases reported as of March 2023. Despite concerns that some cases could be undetected (particularly in the MSM community), potentially preventing outbreak control, the serologic survey identified only 1.3% of MSM patients at high risk for mpox without a known mpox diagnosis who had orthopoxvirus IgM, indicating recent exposure to mpox. That rate of IgM positivity is similar to the 1.4% rate among persons experiencing homelessness in San Francisco during July–October 2022 (*11*). Mpox was retrospectively detected by PCR in 5.6% of lesion swab samples obtained across the country, suggesting that mpox was probably undiagnosed in a small subset of symptomatic patients during the height of the mpox outbreak in the United States. The highest percentage positivity was among those who reported sexual behavior that places someone at increased for STI/HIV. However, the second highest percentage positivity was among those for whom mpox testing was retrospectively ordered by the clinician after negative diagnostic test results for other common rash illnesses, suggesting that clinician awareness was higher for mpox during this period. The data from the 2 analyses reported here indicate that as long as persons are aware of mpox and the need to seek medical care, the percentage of undiagnosed cases remains low, as it did during the peak of the outbreak.

The clinical manifestations (especially skin lesions, pustules, and rashes) of mpox patients can be confused with those of varicella zoster virus and STIs (e.g., herpes and syphilis), and mpox can co-occur with other STIs. However, in the molecular study, we did not find any significant levels of co-infections with mpox and other STIs.

That the earliest positive IgM result was obtained in mid-July suggests infection up to 56 days earlier. The lack of IgM detection before that time, in a small sample from 1 region, is suggestive that cases may not have been prevalent before the first detection on May 17. Of the 3 persons with an IgM-positive result, 2 self-reported symptoms consistent with mpox within the previous 3 months.

Among the limitations of our analyses, the response rates to the survey were low. The serologic survey relied on patient self-screening through the survey questionnaire, self-reported symptoms, and travel history. Also, the serologic survey was conducted in San Francisco, where infrastructure and resources may not be reflective of other geographic locations. Because the serologic survey was a point seroprevalence study, no follow-up testing or interviews were conducted among the participants who were positive for orthopoxvirus IgM; it is unknown whether any participants previously had signs/symptoms that were not reported on the survey or if signs/symptoms ultimately developed. Only 3 specimens were positive for both orthopoxvirus IgG and IgM. For the other 15 IgG-positive/IgM-negative specimens, it is unknown whether the participants had been exposed to orthopoxvirus beyond the IgM detection window or whether they did not self-report previous vaccination (many JYNNEOS vaccination campaigns were ongoing during the study period). We did not collect information on military service, which would include persons who may have received ACAM2000, a live-replicating vaccinia virus vaccine that results in production of orthopoxvirus antibodies. Because we used IgG as the initial screening tool, a participant could have been IgM positive and IgG negative; however, because that window of time is small (3–4 days), the likelihood of missing potential cases is low. The major limitations of molecular testing were similar to those of any study relying on ICD-10-CM codes for analysis and for which detailed patient history was not available beyond the ICD-10-CM codes on test requisitions.

In conclusion, the rate of undiagnosed mpox infections during the peak of reported cases in the United States was low among persons at high risk for disease (represented by participants in the San Francisco serosurvey). Mpox diagnosis was probably missed for some persons with rash (represented by retrospective molecular testing at HealthTrackRx), and providers should remain vigilant and conduct mpox testing from lesion swab samples on patients with mpox signs/symptoms. We rapidly collected our data during the peak of the outbreak to provide information for the epidemiologic response. Ongoing serologic and molecular studies that are underway that use specimens stored before May 2022 will be useful for determining whether mpox was present before the outbreak was identified in the United States.

Appendix 1 Questionnaire used in study of undiagnosed monkeypox virus infections, United States, June–September 2022.

Appendix 2 Additional information for study of undiagnosed monkeypox virus infections, United States, June–September 2022.
