# Supplementary material for: Prevalence of Undiagnosed Monkeypox Virus Infections during Global Mpox Outbreak, United States, June–September 2022
Source: Emerg Infect Dis. 2023 Nov;29(11):2307–14. doi: 10.3201/eid2911.230940 (PMC10617324; doi:10.3201/eid2911.230940)
Supplement: Appendix 1 — Questionnaire used in study of undiagnosed monkeypox virus infections, United States, June–September 2022. [file 23-0940-Techapp-s1.pdf]

Article DOI: https://doi.org/10.3201/eid2911.230940

EID cannot ensure accessibility for supplementary materials supplied by authors. Readers who have difficulty accessing supplementary content should contact the authors for assistance.

Prevalence of Undiagnosed Monkeypox Virus Infections during Global Mpox Outbreak, United States, June–September 2022

Appendix 1

Questionnaire

The following pages show the 7-question electronic survey given to study participants at clinic D who were recruited by a query into the electronic medical record system from HIV and HIV pre-exposure prophylaxis registries. A subset of MSM patients 18–50 years of age were sent an invitation to participate and completed the questionnaire.

Serosurvey

Thank you for your willingness to participate in this evaluation. We will ask you a few questions (< 5 mins) about your activities in the past 3 months. We will ask to look at your arms to see if there is a smallpox vaccine scar and ask for your age. Also, we will take a blood sample of 5 ml (about one teaspoon) from you. We will test your blood to see if you have been exposed to the Orthopoxvirus, Monkeypox virus. We will use an assay to detect the presence of anti-Orthopoxvirus antibodies.

This activity was reviewed by CDC and was conducted consistent with applicable federal law and CDC policy.§

§ See e.g., 45 C.F.R. part 46.102(l)(2), 21 C.F.R. part 56; 42 U.S.C. §241(d); 5 U.S.C. §552a; 44 U.S.C. §3501 et seq.

Would you like to complete this survey in Spanish or English?<br><br>¿Quiere completar esta encuesta en español o inglés?	◯ English/inglés<br>◯ Spanish/español

---

Por favor, siga este enlace para acceder a la encuesta en español: haga clic aquí

Demographics

Have you previously participated in this study?	◯ Yes<br>◯ No
In which clinic are you taking this survey?	◯ San Francisco City Clinic<br>◯ San Francisco AIDS Foundation (Strut)<br>◯ Ward 86<br>◯ Kaiser<br>◯ Other
You mentioned you are located in another clinic, can you please specify?	__________________________________
Participant number	__________________________________
Are you older than 50 years old?	◯ Yes<br>◯ No
What is your age?	__________________________________
What was your sex assigned at birth?	◯ Female<br>◯ Male<br>◯ Prefer not to answer
Do you currently describe yourself as male, female, or transgender?	◯ Female<br>◯ Male<br>◯ Transgender female<br>◯ Transgender male<br>◯ Another gender identity<br>◯ Prefer not to answer
You mentioned you prefer another gender identity, can you specify?	__________________________________
Which of the following best represents how you think of yourself?	◯ Lesbian or gay<br>◯ Straight (not gay or lesbian)<br>◯ Bisexual<br>◯ A different term<br>◯ Prefer not to answer
You mentioned you prefer a different term, can you specify?	__________________________________
What is your race?	☐ American Indian or Alaska Native<br>☐ Asian<br>☐ Black or African American<br>☐ Native Hawaiian or Other Pacific Islander<br>☐ White<br>☐ Other<br>☐ Declined to answer<br>☐ Unknown
Please specify 'Other' race.	__________________________________

What is your ethnicity?	◯ Hispanic or Latino<br>◯ Non-Hispanic or Latino<br>◯ Other<br>◯ Declined to answer<br>◯ Unknown
Please specify 'Other' ethnicity.	__________________________
Have you ever had any male-to-male sexual contact? This includes: genital touching, oral-penile (e.g. gave or received a blowjob), oral-anal (e.g. rimming), and/or penile-anal sex (e.g. insertive or receptive sex as a top, bottom or versatile).	◯ Yes<br>◯ No

Health Information

Survey 25% Complete

Have you previously been vaccinated for smallpox or monkeypox?	◯ Yes<br>◯ No<br>◯ Unknown
Which smallpox vaccine did you receive?	◯ Dryvax (discontinued in 2008)<br>◯ ACAM2000 (started in 2008)<br>◯ JYNNEOS (started in 2022)<br>◯ Unknown
Have you previously received a monkeypox diagnosis?	◯ Yes<br>◯ No
In the past 3 months, have you been sick with something other than a chronic illness?	◯ Yes<br>◯ No
You mentioned you may have been sick in the past 3 months, what symptoms did you have?	☐ Rash<br>☐ Fever<br>☐ Chills<br>☐ Sweats<br>☐ General feeling of illness/weakness<br>☐ Muscle aches<br>☐ Itchiness<br>☐ Headache<br>☐ Eye lesions<br>☐ Red or swollen eyes<br>☐ Runny nose<br>☐ Wheeze<br>☐ Cough<br>☐ Shortness of breath<br>☐ Difficulty breathing in, with high pitched wheezy sound<br>☐ Swollen glands<br>☐ Sore throat<br>☐ Diarrhea<br>☐ Nausea/Vomiting<br>☐ Abdominal pain<br>☐ Back pain<br>☐ Urgency to defecate<br>☐ Rectal pain<br>☐ Rectal bleeding<br>☐ Pus/blood on stool<br>☐ Other symptoms (specify)
Please specify other symptoms:	______________________________
When did these symptoms start?	______________________________
WARNING: The date entered is more than 3 months ago!	
WARNING: The date entered is a future date!	
Are you currently sick with something other than a chronic illness?	◯ Yes<br>◯ No

Close personal contact includes contact with:

Any person presenting similar symptoms OR A known confirmed case OR Material (body fluids, objects, bedding, etc.) that came in contact with a case or a person with similar symptoms

Close intimate contact includes:

Hugging, cuddling, kissing, massaging, other skin-to-skin contact OR Oral, anal, and vaginal sex or touching the genitals (penis, testicles, labia, and vagina) or anus (butt) of another person OR Touching fabrics or objects during sex that were used by another person that have not been disinfected, such as bedding, towels, fetish gear, and sex toys

Have you been in close personal and/or intimate contact with anyone who has received a monkeypox diagnosis?	◯ Yes<br>◯ No
In the past 1 month, how many unique sexual partners have you had? (Sexual partners refers to partners for oral, anal, or vaginal sex.) Please provide your best estimate if you do not know an exact number.	__________________________________
Do you have any known immunocompromising conditions or take immunosuppressive medications? Immunocompromising conditions can include diseases like HIV/AIDS, diabetes, lupus, organ transplants, stem cell transplants, and cancer. Certain medicines like chemotherapy, biologic therapies, and steroids can also weaken the immune system.	☐ Yes - HIV<br>☐ Yes - a condition that's not HIV<br>☐ No
What is you CD4 count, if known?	◯ ≥200<br>◯ < 200<br>◯ Unknown
What is your viral load, if known?	◯ ≥200<br>◯ < 200 or undetectable<br>◯ Unknown
You mentioned that you have an immunocompromising condition or treatment, please describe the associated condition or treatment.	__________________________________
Date of serum sample	__________________________________<br>(@TODAY)

Travel

Survey 50% Complete

Did you travel within the past 3 months out of the state or country?	◯ Yes<br>◯ No
Trip 1: Where did you travel (city, state, country)	__________________
Trip 1: When did you leave?	__________________
Trip 1: When did you return?	__________________
The return date of your trip is before the leave date of your trip	
WARNING: The date entered is a future date!	
WARNING: The date entered is more than 3 months ago!	
Was there another trip?	◯ Yes<br>◯ No
Trip 2: Where did you travel (city, state, country)	__________________
Trip 2: When did you leave?	__________________
Trip 2: When did you return?	__________________
The return date of your trip is before the leave date of your trip	
WARNING: The date entered is a future date!	
WARNING: The date entered is more than 3 months ago!	
Was there another trip?	◯ Yes<br>◯ No
Trip 3: Where did you travel (city, state, country)	__________________
Trip 3: When did you leave?	__________________
Trip 3: When did you return?	__________________
The return date of your trip is before the leave date of your trip	
WARNING: The date entered is a future date!	

WARNING: The date entered is more than 3 months ago!

Was there another trip?

- ◯ Yes
- ◯ No

Trip 4: Where did you travel (city, state, country) __________________________________

Trip 4: When did you leave? __________________________________

Trip 4: When did you return? __________________________________

The return date of your trip is before the leave date of your trip

WARNING: The date entered is a future date!

WARNING: The date entered is more than 3 months ago!

Events

Survey 75% Complete

In the past 3 months, have you attended any large public or private events (e.g., festivals, parades, weddings, clubs, sex parties)?
◯ Yes
◯ No

What was the name of this event? ______________________

When did this event take place? ______________________

WARNING: The date entered is a future date!

WARNING: The date entered is more than 3 months ago!

Where did this event take place (city, state, country)? ______________________

Was there another event?
◯ Yes
◯ No

What was the name of this event? ______________________

When did this event take place? ______________________

WARNING: The date entered is a future date!

WARNING: The date entered is more than 3 months ago!

Where did this event take place (city, state, country)? ______________________

Was there another event?
◯ Yes
◯ No

What was the name of this event? ______________________

When did this event take place? ______________________

WARNING: The date entered is a future date!

WARNING: The date entered is more than 3 months ago!

Where did this event take place (city, state, country)? ______________________
