# Supplementary material for: Prevalence of Undiagnosed Monkeypox Virus Infections during Global Mpox Outbreak, United States, June–September 2022
Source: Emerg Infect Dis. 2023 Nov;29(11):2307–14. doi: 10.3201/eid2911.230940 (PMC10617324; doi:10.3201/eid2911.230940)
Supplement: Appendix 2 — Additional information for study of undiagnosed monkeypox virus infections, United States, June–September 2022. [file 23-0940-Techapp-s2.pdf]

Article DOI: https://doi.org/10.3201/eid2911.230940

EID cannot ensure accessibility for supplementary materials supplied by authors. Readers who have difficulty accessing supplementary content should contact the authors for assistance.

Prevalence of Undiagnosed Monkeypox Virus Infections during Global Mpox Outbreak, United States, June–September 2022

Appendix 2

Appendix Table. ICD-10-CM codes with possible symptom and risk factor overlap with mpox.

ICD Code	Concept name
A51.0	Primary genital syphilis
A53.9	Syphilis unspecified
B00	Herpesviral (herpes simplex) infections
B00.9	Herpesviral infection unspecified
B02	Herpes zoster
L98.9	Disorder of skin and subcutaneous tissue, unspecified
L08.89	Other specified local infections of the skin and subcutaneous tissue
N00-N99	Diseases of genitourinary system (nonspecific)
N48.5	Ulcer of the penis
N48.89	Other specified disorders of the penis
N50.9	Disorder of male genital organs unspecified
N76.5	Ulceration of vagina
N76.6	Ulceration of vulva
N89.9	Noninflammatory disorder of vagina unspecified
N90	Other noninflammatory disorders of vulva and perineum
N90.89	Other spec noninflammatory disorder of the vulva and perineum
N90.9	Noninflammatory disorder vulva and perineum unspecified
R21.0	Rash and other nonspecific skin eruption
R23.8	Other skin changes
Z72.51	High-risk heterosexual behavior
Z72.52	High-risk homosexual behavior
Z75.52	High risk bisexual behavior
